# Diagnostic accuracy of late gadolinium enhancement cardiac MRI for coronary artery disease in patients with reduced left ventricular ejection fraction

**DOI:** 10.1136/heartjnl-2024-325419

**Published:** 2025-03-27

**Authors:** Louis-Marie Desroche, Arthur Darmon, Yoan Lavie-Badie, Damien Mandry, Gregory Ducrocq, Thiziri Si-Moussi, Isabelle Durand-Zaleski, Damien Millischer, Olivier Milleron, Olivier Huttin, Mathieu Valla, Lionel Mangin, Bruno Farah, Christelle Diakov, Damien Logeart, Benjamin Safar, Jean-Yves Travers, Jules Mesnier, Alexandra Vappereau, Toni Alfaiate, Charles Burdet, Guillaume Jondeau, on behalf of the CAMAREC investigators, Olivier Nallet

**Affiliations:** 1 Cardiology Department, La Réunion University Hospital, Saint-Denis, France; 2 CIC-EC INSERM1410, La Réunion University Hospital, Saint-Denis, France; 3 Cardiology Department, Centre Cardiologique du Nord, Saint-Denis, France; 4 Cardiology Department, Toulouse University Hospital, Toulouse, France; 5 Radiology Department, Nancy University Hospital, Nancy, France; 6 Bichat - Claude-Bernard Hospital Cardiology Service, Paris, France; 7 INSERM U1148 LVTS, Bichat Hospital, Paris, France; 8 French Alliance for Cardiovascular Trials (FACT), Paris, France; 9 Paris University, Paris, France; 10 Clinical Research Unit-Health Economics (URC-Eco), APHP, Paris, France; 11 INSERM 1153 CRESS Research Center in Epidemiology and Statistics, Sorbonne Paris Cité, Paris, France; 12 Cardiology Department, Montfermeil Hospital, Montfermeil, France; 13 Cardiology Department, Nancy University Hospital, Nancy, France; 14 INSERM U1116, Nancy University Hospital, Nancy, France; 15 Lorraine University, Nancy, France; 16 CIC-1433, Nancy Hospital, Nancy, France; 17 Cardiology Department, Mercy Hospital, CHR Metz-Thionville, Metz, France; 18 Cardiology Department, Centre Hospitalier Annecy Genevois, Epagny Metz-Tessy, France; 19 Cardiology Department, Pasteur Clinic, Toulouse, France; 20 Cardiology Department, Institut Mutualiste Montsouris, Paris, France; 21 Cardiology, Universitary Hospital Saint-Louis – Lariboisière – Fernand-Widal, AP-HP, Paris, France; 22 Hopital Montfermeil, Montfermeil, France; 23 Radiology Department, La Réunion University Hospital, Saint-Denis, France; 24 Department of Epidemiology, Biostatistics and Clinical Research, Bichat Hospital, Paris, France; 25 CIC 1425, Bichat Hospital, Paris, France

**Keywords:** Coronary artery disease, Magnetic Resonance Imaging, Heart Failure, Systolic, Health Care Economics and Organizations

## Abstract

**Background:**

Identifying significant coronary artery disease (CAD) in patients with reduced left ventricular ejection fraction (rLVEF) is essential for guiding therapeutic decisions, including medical management, device implantation and potential revascularisation. Prior studies suggested that rest cardiac MRI (CMR) with late gadolinium enhancement (LGE) could reliably detect significant CAD. We aimed to evaluate the diagnostic accuracy of rest LGE-CMR for predicting significant CAD in rLVEF patients.

**Methods:**

In this prospective, multicentre cohort study across 10 centres, adults with new-onset rLVEF≤45% without obvious cause were included. All patients underwent rest CMR and coronary angiography. Independent, blinded committees reviewed images. Significant CAD was defined as ≥70% stenosis in major coronary arteries. Ischaemic scars were identified on CMR as subendocardial LGE. The primary outcome was the sensitivity of CMR in detecting significant CAD.

**Results:**

Among 380 patients (median age 63 years, 68% male), significant CAD was present in 49 (13%). CMR identified ischaemic scars in 106 (28%). The sensitivity of CMR for detecting significant CAD was 57% (95% CI: 43% to 71%), specificity 76% (95% CI: 72% to 81%), positive predictive value 26% (95% CI: 18% to 35%) and negative predictive value 92% (95% CI: 89% to 95%). A CMR-first strategy would have missed 43% of significant CAD cases, many requiring revascularisation (86% of missed cases).

**Conclusions:**

In this large, prospective multicentre study with independent image review, rest LGE-CMR demonstrated limited sensitivity for detecting significant CAD in patients with rLVEF. Relying solely on CMR could lead to missed diagnoses and undertreatment. CMR should be integrated with other diagnostic tools to optimise care in this population.

**Trial registration number:**

NCT03231189.

**WHAT IS ALREADY KNOWN ON THIS TOPIC**

- Cardiac MR with late gadolinium enhancement (LGE-CMR) at rest is widely used to differentiate ischaemic from non-ischaemic cardiomyopathies. Previous small studies suggested it could reliably predict significant coronary artery disease (CAD) in patients with reduced left ventricular ejection fraction (rLVEF).

**WHAT THIS STUDY ADDS**

- This large, prospective multicentre study found that rest LGE-CMR has limited sensitivity (57%) for detecting significant CAD in patients with rLVEF, indicating that relying solely on this technique could miss 43% of significant CAD cases.

**HOW THIS STUDY MIGHT AFFECT RESEARCH, PRACTICE OR POLICY**

- The findings suggest that rest LGE-CMR should not be used alone to exclude significant CAD in patients with rLVEF. A multimodal diagnostic approach, integrating CMR with other imaging modalities, is essential to optimise patient care and avoid missed diagnoses.

## Introduction

 Heart failure with reduced left ventricular ejection fraction (rLVEF) is a prevalent and increasing global concern, with coronary artery disease (CAD) being a leading cause.^1^ Accurately identifying ischaemic heart failure is crucial for determining prognosis^2 3^ and guiding evidence-based interventions, including pharmacological treatments, defibrillator implantation and timely revascularisation when appropriate.^4^,^6^ The invasive nature of coronary angiography (CA) highlights the need for non-invasive, accurate and accessible diagnostic methods.

Non-invasive stress testing still struggles to accurately rule out CAD in rLVEF even when surgical revascularisation is required.^7^,^9^ Stress cardiac MR (CMR) has shown promise in this context; however, its clinical adoption is limited by factors such as availability, cost and variability in interpretation.^10^ Moreover, debates persist regarding the incremental value of stress CMR over the use of late gadolinium enhancement (LGE) imaging alone.^11 12^

In current guidelines, LGE-CMR has emerged as a valuable tool for differentiating ischaemic from non-ischaemic cardiomyopathies. ^5 13^ Early studies suggested that the presence of subendocardial LGE could reliably predict significant CAD in patients with rLVEF.^1214^,^17^ It has been hypothesised that rLVEF associated with prognostically significant CAD is strongly correlated with the presence of ischaemic scarring in at least one myocardial segment.^15^ With CMR routinely recommended for the assessment of patients with rLVEF,^4 5^ there is potential to streamline patient care by using CMR to both evaluate myocardial function and exclude significant CAD.

However, these previous studies have limitations like small sample sizes, single-centre retrospective designs and the lack of independent review committees, limiting generalisability. Furthermore, a recent large-scale retrospective study highlighted a considerable incidence of non-ischaemic or mixed cardiomyopathies in patients with significant CAD,^18^ casting doubt on the predictive reliability of LGE-CMR for detecting significant CAD.

The CAMAREC study aims to rigorously evaluate the diagnostic accuracy of LGE-CMR in identifying significant CAD in rLVEF patients across multiple centres and to assess the cost-effectiveness of a CMR-first diagnostic strategy.

## Methods

### Study design

The CAMAREC study was a prospective, multicentre cohort study across 10 French centres, assessing CMR diagnostic performance for predicting significant CAD in adults (≥18 years) with new and unexplained rLVEF (≤45%), compared with CA as the gold standard. To address the pragmatic question of the effectiveness of CMR in excluding CAD and given the limited existing evidence level, a non-randomised design was selected as the most suitable approach. The LVEF≤45% threshold was determined based on prior studies and guidelines available at the time of study conception.^4 19^ Details of the study design have been published previously.^20^ Patients aged ≥18 years with a recent (≤8 weeks) rLVEF diagnosis, without an obvious cause (eg, arrhythmia, severe valvulopathy, angina or known CAD), were included. Evidence of ischaemic cardiomyopathy on ECG or echocardiography did not exclude them. Key exclusions were contraindications to CA or CMR and pregnancy or breastfeeding (online supplemental table 1). Participants underwent rest-CMR followed by CA within 2 weeks, with blinded committees reviewing each modality.

The primary endpoint was the sensitivity of the presence of ischaemic scar on CMR for the diagnosis of significant CAD on CA (CA+). Secondary endpoints included specificity, positive predictive value (PPV), and negative predictive value (NPV) of CMR for predicting CA+ patients. Our analysis included a medicoeconomic analysis for which the endpoints were the incremental cost-effectiveness ratio (ICER) of a ‘CMR-first strategy’, using diagnostic performance and the decremental cost per additional false negative. The ‘CMR-first strategy’ was defined as a diagnostic strategy in which all patients would undergo CMR first and CA only in the presence of ischaemic scar on CMR.

This study adheres to the Standards for Reporting of Diagnostic Accuracy (STARD) guidelines. It has been registered in the ClinicalTrials.org database (NCT03231189). Patients or the public were not involved in the design, conduct, reporting or dissemination plans of this research.

### Procedures

The CMR multicentric protocol was designed to be feasible across diverse settings to reflect real-life scenarios. Images were acquired in continuous short-axis positions encompassing the left ventricle during a brief breath-hold, using a cine steady-state free precession sequence. LGE images were acquired approximately 10 min after injection of a gadolinium-based contrast agent. The primary LGE sequences used were a 3D inversion recovery gradient echo-sequence, aligned with the slice positions of the cine images. While phase-sensitive inversion recovery or’“PSIR’ sequences were also permissible, the number and location of slices were not predetermined to echo real-world clinical settings. While no standardised table was provided for pulse sequence details due to the diversity of centres and equipment, adherence to the SCMR 2020 guidelines^21^ was ensured at each local protocol. Although this was optional in the CMR protocol, T1 mapping was frequently performed.

CA was performed according to the routine clinical practice of each recruiting centre. The decision to perform myocardial revascularisation, whether by percutaneous coronary intervention (PCI) or coronary artery bypass grafting (CABG) was left to the operator’s discretion, based on clinical, angiographic and physiological assessment.

Centralised and independent committees blindly reviewed and adjudicated both CMR and CA. Both review committees were composed of two experts in their field in university hospitals (DMan and YL-B for CMR, GD and JM for CA). In case of disagreement between the two experts, the opinion of a third expert was sought.

### Definition of CMR+ and CA+

Patients were considered to have an ischaemic scar on CMR (CMR+) if LGE involved the subendocardium and was observed in at least one left ventricular segment, irrespective of the presence of additional non-ischaemic LGE patterns. Otherwise, they were considered CMR−. This definition aligns with prior studies, ensuring comparability of results.^1214^,^16^

Patients were considered to have ‘significant CAD’ on CA (CA+), according to Felker’s prognostic criteria^2^ adapted from ESC guidelines^22^ if at least one of the following criteria of coronary artery stenosis was present:

1. >70% stenosis of the left main coronary artery.
2. >70% stenosis of the proximal left anterior descending artery.
3. ≥2 other proximal vessels with >70% stenosis (ie, mid-left anterior descending artery, right coronary artery for right main disease and left circumflex artery for left main disease).

Otherwise, they were considered CA−.

### Statistical analysis

The sample size was determined based on the sensitivity of CMR to predict CA+, with the aim of limiting the false negative rate. In our retrospective pilot study,^17^ the sensitivity was estimated to be 96%, with a prevalence of significant CAD in the population of 17%. To ensure a statistical precision of 5% for sensitivity, a sample of 353 patients was required. This number was increased to 406 patients to account for a 15% rate of unusable data.

The definition of sensitivity, specificity, PPV and NPV is detailed in online supplemental table 2. 95% CIs were calculated using the binomial distribution.

In order to better understand the differences between patients classified as true positive or false negative for CMR, we compared their clinical and CMR characteristics using non-parametric Wilcoxon or Fisher’s exact test, as appropriate. Patient characteristics were described using frequency and percentage for categorical variables, and median and IQR values for continuous variables. Analyses included all available data without imputing missing values, using SAS software V.9.4.

The health economic evaluation, adhering to the French national health authority^23^ and literature,^24^ compared CMR-first strategy costs to systematic CA, based on patient outcomes and required subsequent CA. A sensitivity analysis explored the systematic use of CMR added to CA as a first-line diagnostic strategy, as CMR is increasingly being performed. All procedure and hospitalisation costs were derived from the French Healthcare System (online supplemental table 3). The analysis tested a decrementally cost-effective hypothesis for CMR-first strategy against the CA gold standard, focusing on diagnostic accuracy. ICERs were computed from cost differences and diagnostic accuracy, incorporating complications costs. Missing data on hospital stay were replaced by the mean according to the type of patient diagnosis (true negative, true positive, false negative). We assumed that there would be no false positives in the CMR-first strategy (such as patients with a Myocardial Infarction with Non-Obstructive Coronary Artery -MINOCA), as CMR+ patients would subsequently undergo CA, ensuring that they would be correctly identified as true negatives. Bootstrapping provided 95% CIs, with R (V.4.01) for analysis. All costs are reported in euros and reflect the French social health insurance schedule and hospital cost accounting for the year 2023 (online supplemental table 3).

## Results

### Patient enrolment and characteristics

**Figure 1:**
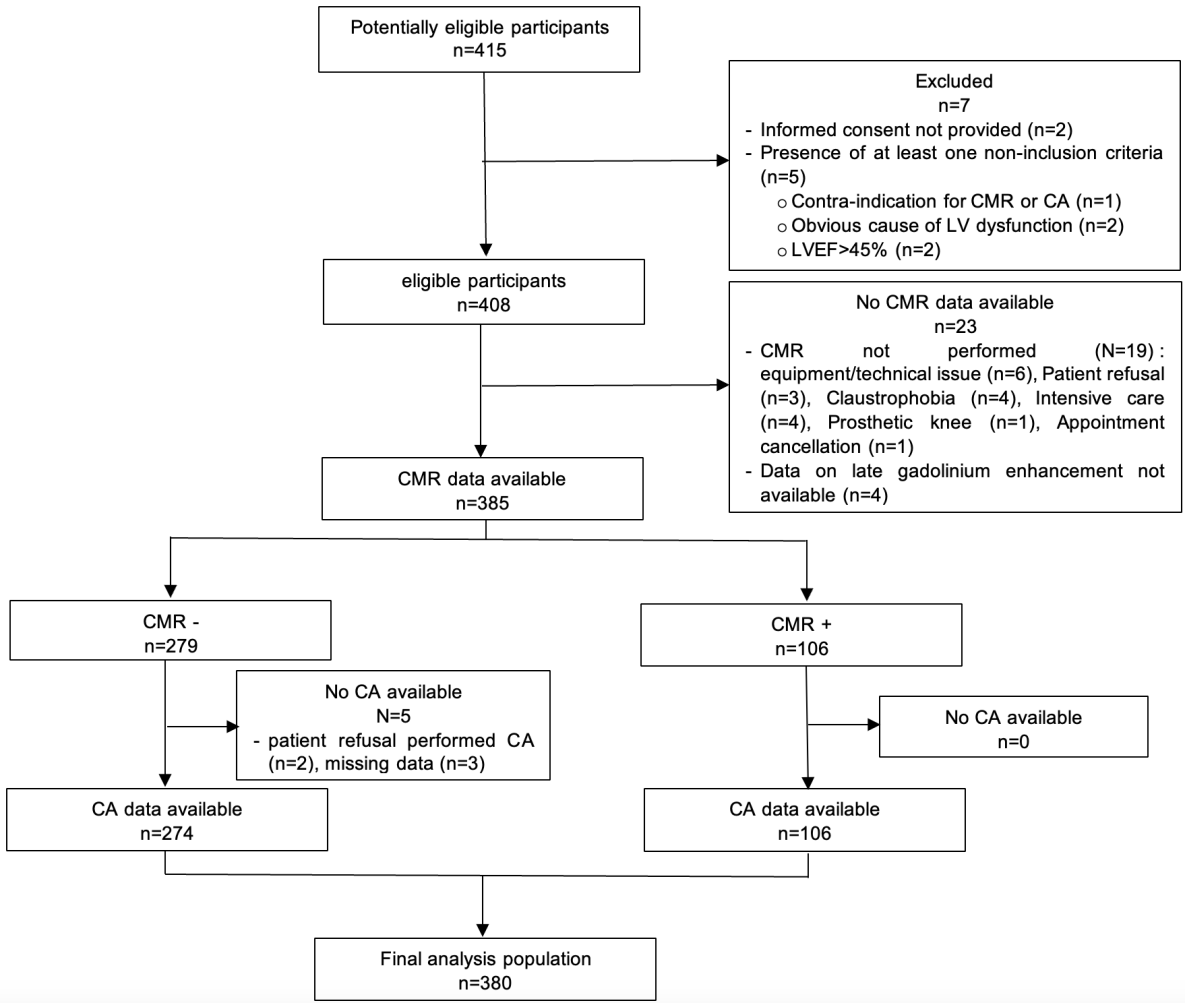
Flow chart of the study. CA, coronary angiography; CMR, cardiac MR; LVEF, left ventricular ejection fraction.

From May 2018 to August 2021, a total of 415 patients were screened, with 408 (98%) meeting the inclusion criteria. Of these, both CA and CMR data were available for 380 patients (93%), which formed the final cohort for primary analysis (figure 1). There were no significant adverse events reported during the performance of either CMR or CA.

Baseline characteristics of the patients are presented in table 1. The median age was 63 years (IQR: 52.0–71.0), and 68% (n=259) were male. Common cardiovascular risk factors included hypertension (42%), smoking (53%), diabetes (22%) and dyslipidaemia (19%). Most patients (56%) were in New York Heart Association functional class III or IV, and dyspnoea was the leading symptom (62%). The median LVEF was 28% by echocardiography and 27% by CMR, with 47% of patients demonstrating right ventricular systolic dysfunction by CMR.

**Table 1:** Baseline characteristics of the study population

	Overall N=380
Age, median (IQR), years	63 (52–71)
Male sex, n (%)	259 (68)
Body mass index, median (IQR)	26 (22–30)
Cardiovascular risk factors	
Hypertension, n (%)	158 (42)
Dyslipidaemia, n (%)	73 (19)
Smoking history, n (%)	197 (53)
Diabetes, n (%)	85 (22)
Family history of premature cardiovascular disease, n (%)	46 (12)
Past medical history	
Atrial fibrillation, n (%)	26 (7)
Peripheral artery disease, n (%)	21 (6)
History of chemotherapy, n (%)	37 (10)
Clinical condition	
NYHA I/II, n (%)	153 (44)
NYHA III/IV, n (%)	197 (56)
Dyspnoea as the leading symptom, n (%)	232 (62)
Biology	
Creatinine, median (IQR), mg/dL	10(8–12)
NTproBNP, median (IQR), mg/dL	1757 (999–3791)
Echocardiography data	
Left ventricular ejection fraction, median (IQR), %	28 (20–35)
Left ventricular indexed end-diastolic volume, median (IQR), mL/m^2^	90 (70–109)
Right ventricular systolic dysfunction, n (%)	113 (30)
Pulmonary artery systolic pressure, median (IQR), mm Hg	35 (30–46)
Cardiac magnetic resonance data	
Left ventricular ejection fraction, median (IQR), %	27 (20–35)
Left ventricular end-diastolic volume, median (IQR), mL	228 (180–293)
Right ventricular systolic dysfunction, n (%)	175 (47)
Right ventricular end-diastolic volume, median (IQR), mL	189 (151–238)
Late gadolinium enhancement	
No late gadolinium enhancement	214 (56)
Subendocardial or transmural	106 (28)
Mid-myocardial	29 (8)
Subepicardial	21 (6)
Mid-myocardial and subepicardial	10 (3)
Coronary angiogram data	
Presence of at least one epicardial stenosis >50%, n (%)	148 (39)
Presence of significant coronary artery disease, n (%)	49 (13)

The percentages are calculated based on the number of patients with available data for each variable.

NT-proBNP, N-terminal pro b-type natriuretic peptide; NYHA, New York Heart Association.

### CMR and CA results and revascularization

Of the 380 patients, CMR images were acquired using 1.5T systems in 203 (54%) patients and 3T systems in 164 (43%), with 13 (3%) cases lacking system data. Image quality was suboptimal in 81 cases (22%), with a higher prevalence of suboptimal quality observed in discordant interpretations between local and central readings (32.1% vs 19.9%, p=0.047). Subendocardial or transmural LGE was detected in 106 patients (28%), classifying them as CMR+. Conversely, all the other patients were CMR–, with 214 patients (56%) without any enhancement, and 60 patients (16%) with non-subendocardial LGE patterns (mid-myocardial, subepicardial or mixed). CMR results influencing patient management (by LGE pattern, viability assessment, thrombus detection, etc) concerned 38% of patients and are detailed in online supplemental table 4.

Significant CAD (CA+) was confirmed in 49 patients (13%) via CA. Of these, 31 patients (63%) had >70% stenosis in the proximal left anterior descending artery, 22 (45%) had multivessel disease and 4 (8%) had a >70% stenosis on the left main coronary artery.

The concordance between local centre interpretations and the adjudication committee was substantial for both CMR and CA. For CMR interpretations, the kappa coefficient was 0.64 (95% CI: 0.553 to 0.728), with concordant interpretations in 325 out of 379 cases (85.7%). For CA, the kappa coefficient was 0.70 (95% CI: 0.595 to 0.804), with concordant interpretations in 368 out of 396 cases (92.9%) (online supplemental table 5).

Revascularisation (PCI or CABG) was performed in 86% of these cases. No significant differences were observed in revascularisation rates between CMR+and CMR− groups (online supplemental table 6).

### Diagnostic performance of CMR to predict CA+

Among the 380 patients, 253/380 patients (67%) were CMR−/CA−, 78 (21%) were CMR+/CA−, 28 (7%) were CMR+/CA+ and 21 (5%) were CMR−/CA+. The sensitivity of CMR to predict CA+ patients was 57% (95% CI: 43% to 71%), specificity 76% (95% CI: 72% to 81), PPV 26% (95% CI: 18% to 35%) and NPV 92% (95% CI: 89% to 95%) (table 2).

**Table 2:** Performance characteristics of CMR for predicting significant coronary artery disease

(A)	CA−	CA+	Total
CMR−	253	21	274
CMR+	78	28	106
**Total**	331	49	380

(B)		
Test performance metric	Value (%)	95% CI
Sensitivity	57	43 to 71
Specificity	76	72 to 81
Positive predictive value	26	18 to 35
Negative predictive value	92	89 to 95

Contingency table comparing CMR results with CA results. CMR− denotes patients with a negative CMR result, while CMR+ indicates a positive CMR result. CA− represents patients without significant coronary artery disease on angiography and CA+ represents those with significant coronary artery disease.

CA, coronary angiography; CMR, cardiac MR.

Additional sensitivity analyses (eg, excluding patients with suboptimal image quality, considering only the interventional cardiologist opinion for defining CA+ instead of Felker’s criteria, considering different LVEF subgroups or adding first pass perfusion and T1 mapping) did not substantially alter the diagnostic performance of CMR (online supplemental table 7).

Notably, among the 21 false-negative cases (CMR−/CA+), 76% had stenosis >70% in the proximal segment of the left anterior descending coronary artery, 33% had >70% stenosis in two epicardial vessels and 10% had >70% stenosis of the left main coronary artery. This group had a high rate of revascularisation (86%), comparable to the overall CA+ population. Several CA images of CMR−/CA+ patients are shown in online supplemental figure 1.

### Comparison between false-negative and true-positive CMR cases

Further analysis revealed significant differences between the true-positive (CMR+CA+) and false-negative (CMR-CA+) groups (table 3). The CMR-CA+group was older, with a median age of 70 years compared with 64 years in the CMR+CA+ group (p=0.0152). Additionally, the CMR+CA+ group had significantly higher troponin I levels and a significantly greater number of affected coronary vessels. Non-subendocardial enhancement patterns (centromyocardial or subepicardial) were more common in the CMR−/CA+ group, though these last differences did not reach statistical significance.

**Table 3:** Comparison of true-positive (CMR+CA+) and false-negative (CMR-CA+) groups

Characteristic	Total (N=49)	CMR-CA+ N=21	CMR+CA+ N=28	P value
Age, median (IQR), years	66.0 (58.0–74.0)	70.0 (65.0–82.0)	64.0 (55.0–72.0)	0.02
Troponin I (µg/L), median (IQR)	0.3 (0.1–0.5)	0.1 (0.0–0.3)	0.4 (0.2–0.7)	0.02
Non-subendocardial LGE				
Centromyocardial, n (%)	8 (16.3%)	6 (28.6%)	2 (7.1%)	0.06
Subepicardial, n (%)	6 (12.2%)	4 (19.0%)	2 (7.1%)	0.38
Number of vessels involved				
1 vessel, n (%):	10 (20.4%)	6 (28.6%)	4 (14.3%)	0.03
2 vessels, n (%):	19 (38.8%)	11 (52.4%)	8 (28.6%)	
3 vessels, n (%):	20 (40.8%)	4 (19.0%)	16 (57.1%)	

Additional exploratory variables (with smallest p=0.053) included sex distribution, presence of hypertension, dyslipidaemia, history of smoking, diabetes, family history of cardiovascular disease, BMI, atrial fibrillation, presence of cardiotoxicity (eg, alcoholism, HIV), peripheral artery disease, Troponin T, echocardiographic measures (eg, left ventricular ejection fraction, diameters, volumes), right ventricular function, T1 mapping values, ECG findings, location of the lesions and are presented in online supplemental table 10.

BMI, body mass index; CA, coronary angiography; CMR, cardiac MR; LGE, late gadolinium enhancement.

### Cost-effectiveness of CMR-first strategy

A cost-effectiveness analysis compared a ‘CMR-first’ strategy to a ‘CA-first’ approach. The CMR-first strategy could save an average of €586 (95% CI −€71 to €1224) per patient, but it would miss 43% of significant CAD cases. The ICER highlights this balance, with €7089 (95% CI €2445 to €21 949) saved per missed CAD diagnosis, though this cost-saving could result in missed treatments for some patients. Further details, including comprehensive cost-effectiveness analyses and ICER calculations, are available in online supplemental table 8 and online supplemental figure 2.

## Discussion

### Key finding

In this large, prospective, multicentre study involving 380 patients across 10 centres, we found that rest LGE-CMR imaging demonstrated limited sensitivity (57% (95% CI: 43% to 71%)) for detecting significant CAD in patients with rLVEF), contrary to the previous studies.^1214^,^17^ While a CMR-first diagnostic strategy could potentially reduce healthcare costs, it would have missed 43% of significant CAD cases in our cohort, potentially delaying or depriving patients of critical interventions such as optimal medical therapy, defibrillator implantation and revascularisation when indicated. Even after controlling for image quality, alternative CAD definitions, T1 mapping and subgroup analyses, the sensitivity remained lower than expected. These findings suggest that relying solely on rest LGE-CMR to exclude significant CAD in patients with rLVEF may be insufficient.

### Comparisons with previous studies

Our results partially contradict earlier studies that reported higher sensitivity (86%–96%) and specificity (85%–94%) of rest LGE-CMR for predicting significant CAD in patients with rLVEF.^12 14 15 17^ Soriano and Valle-Muñoz additionally reported a strong correlation between the extent of LGE and CAD severity. Thompson *et al* found a perfect sensitivity (100%) but low specificity (44%).^16^ However, these previous studies were generally limited by smaller sample sizes, single-centre retrospective designs, and by the lack of independent blinded review committees for CMR and CA. While our angiographic criteria were generally consistent with previous studies, some used less strict thresholds, such as 50% for the left main.^12 16^ Applying these criteria would have further lowered our sensitivity. Our CMR criteria were also consistent with previous studies, focusing on subendocardial LGE in at least one segment, irrespective of other patterns.

These CMR criteria are designed to predict significant CAD, not to classify cardiomyopathies. Ischaemic cardiomyopathy often involves several segments, aligned with coronary artery territories, whereas most patients with non-ischaemic cardiomyopathy have either no LGE or a patchy centromyocardial pattern not related to the territory of a coronary artery.^25^ As an illustration, Assomull *et al*^26^ reported successful detection of "ischaemic cardiomyopathy" using CMR, but their findings relied on expert opinions that incorporated CMR results. Consequently, some patients with significant CAD were classified as ‘non-ischaemic’ due to the absence of CMR-detected infarction. In contrast, we used predefined angiographic criteria associated with patient prognosis and management^2 3 6^ to define significant CAD independently of CMR results.

### Interpretation of findings

Several factors may explain the limited sensitivity of rest LGE-CMR observed in our study. First, rest LGE-CMR primarily detects myocardial fibrosis as a downstream consequence of significant coronary artery stenosis. However, it has the potential to miss hibernating myocardium downstream of a stenosis causing myocardial dysfunction without scar tissue detectable by LGE. This intrinsic limitation of LGE-CMR may help to explain its high specificity but limited sensitivity for significant CAD detection, as observed in our study and in previous studies,^12 14 15^ highlighting why it may not be suitable as a standalone screening tool for significant CAD in patients with rLVEF, where minimising false negatives is critical.

Second, patients may have mixed cardiomyopathy. Indeed, differences between true-positive (CMR^+^CA^+^) and false-negative (CMR⁻CA^+^) groups suggest heterogeneity in the CA+ patient population. Patients in the false-negative group were older, with significantly less severe CAD, and had a tendency towards non-subendocardial LGE patterns, hinting at concomitant non-ischaemic cardiomyopathies coexisting with significant CAD. This complexity reflects the real-world scenario where CAD and non-ischaemic cardiomyopathies often overlap,^18^ complicating the diagnostic process.

### Clinical implications

These findings highlight the importance of a multimodal diagnostic approach for evaluating significant CAD in patients with rLVEF. The limitations of stress imaging in patients with multivessel disease or diffuse subendocardial ischaemia^7 8 11^ support the need to combine CMR with other non-invasive imaging modalities. Coronary CT angiography (CCTA) is a reliable, non-invasive alternative to CA, with high diagnostic accuracy.^27^ Advances like photon-counting CT further enhance its precision, even in challenging cases with severe calcifications or stents.^28^ Although the IMAGE-HF 1C trial found no significant cost advantage of CCTA over CA in rLVEF, it demonstrated its potential to reduce unnecessary procedures with comparable outcomes.^29^ Furthermore, delayed enhancement by multidetector-CT provides a valuable myocardial assessment offering a comprehensive evaluation of rLVEF.^30^

### Strengths and limitations

A major strength of our study is its prospective, multicentre design, involving a large patient cohort that enhances the generalisability of our findings across diverse clinical settings. The use of independent, blinded adjudication committees for both CMR and CA reduces potential bias.

The interobserver agreement between local interpretations and the adjudication committee was substantial for both CMR and CA, with kappa coefficients of 0.64 and 0.70, respectively. This indicates a good level of consistency in imaging interpretations across different centres and observers.^31^ However, the less-than-perfect agreement highlights inherent variability in imaging interpretation, potentially influenced by suboptimal image quality, underscoring the need for standardised training and protocols to enhance consistency.

The sensitivity difference with our pilot study (96% vs 57%) likely stems from methodological and population differences. While a higher precision (5% vs 14%) would require a larger sample, the 95% CI upper limit of sensitivity (71%) supports the robustness of our conclusions, as 71% would not have been satisfactory either.

The decision to use rest-CMR instead of stress-CMR in our study may be viewed as a limitation. However, some data suggest that, in this specific context, it may not provide additional value over LGE.^11 12^ Furthermore, the objective of this study was to validate the performance of a promising, readily available, inexpensive and reproducible test for a frequent scenario.

The non-randomised design may introduce subject selection bias, although the inclusion of consecutive patients aimed to minimise this risk. Our cost-effectiveness analysis was conducted within the French healthcare system and may not fully capture the economic implications in other contexts. Furthermore, without long-term follow-up data on patient outcomes, the clinical implications of our findings, while suggestive, remain to be fully established.

## Conclusions

In conclusion, our study demonstrates that rest LGE-CMR has limited sensitivity for detecting significant CAD in patients with rLVEF, underscoring the need for a multimodal diagnostic approach. While LGE-CMR remains valuable for identifying non-ischaemic cardiomyopathies, integrating it with other imaging techniques seems essential to improve diagnostic accuracy and patient outcomes, warranting further research to validate these strategies for safe and cost-effective care in this population.

## Supplementary material

10.1136/heartjnl-2024-325419 online supplemental file 1

## Data Availability

Data are available on reasonable request.
