# Supplementary material for: Diagnostic accuracy of late gadolinium enhancement cardiac MRI for coronary artery disease in patients with reduced left ventricular ejection fraction
Source: Heart. 2025 Mar 27;111(20):e325419. doi: 10.1136/heartjnl-2024-325419 (PMC12505035; doi:10.1136/heartjnl-2024-325419)
Supplement: online supplemental file 1 [file heartjnl-111-20-s001.docx]

### Supplemental Appendix

- **Supplemental Table 1:** Inclusion and Non-Inclusion Criteria
- **Supplemental Table 2:** Definition of sensitivity, specificity, positive predictive and negative predictive values
- **Supplemental Table 3:** Financial Comparison of Cardiac MRI and Coronary Angiography Procedures
- **Supplemental Table 4:** Additional CMR Findings Influencing Patient Management
- **Supplemental Table 5:** Interobserver Agreement Between Local Interpretation and Adjudication Committee
- **Supplemental Table 6:** Coronary Artery Disease Details and Revascularization of CA+ Patients
- **Supplemental Table 7:** Sensitivity of CMR in Predicting CA+ under Various Conditions
- **Supplemental Table 8:** Cost-Effectiveness Analysis of CMR-first strategy
- **Supplemental Table 9:** The CAMAREC Co-Investigators
- **Supplemental Table 10:** Comparative Characteristics of CMR-CA+ and CMR+CA+ Patients
- **Supplemental Figure 1:** Examples of Patients with Significant CAD on CA Without Subendocardial Late Gadolinium Enhancement on CMR
- **Supplemental Figure 2:** Cost-Effectiveness Analysis of CMR-First Strategy

## Supplemental Table 1. Inclusion and non-inclusion criteria

| Inclusion Criteria | Non-Inclusion criteria |
| --- | --- |
| (1) aged 18 years or older;  (2) LVEF ≤ 45% on echocardiography;  (3) provided informed consent;  (4) underwent a preliminary clinical examination to exclude obvious etiologies | (1) known significant coronary artery stenosis (history of myocardial infarction or coronary artery stenosis);  (2) formal indication for CA other than LV dysfunction (typical angina, acute coronary syndrome, etc.);  (3) obvious etiology for LV dysfunction (valvular, rhythmic, etc.);  (4) pregnancy, desire for pregnancy, breastfeeding, woman of reproductive age without effective contraception or negative pregnancy test;  (5) other contraindication for cardiac magnetic resonance (CMR) (known severe allergy to gadolinium) or coronary artery angiography;  (6) first diagnosis of LVEF dysfunction > 8 weeks;  (7) patient not covered by social security or the CMU;  (8) patients under guardianship or unable to give consent;  (9) patients already included in another study at the same time;  (10) individuals specifically protected by French law (e.g., those deprived of liberty by administrative or judicial decision, hospitalized without consent, admitted to health and social institution for purposes other than research, minors, and adults who are protected or unable to express their consent). |

LVEF: Left Ventricular Ejection Fraction; CA: Coronary Angiography; LV: Left Ventricular; CMR: Cardiac Magnetic Resonance; CMU: Couverture Maladie Universelle (Universal Health Coverage in French)

## Supplemental Table 2. Definition of sensitivity, specificity, positive predictive and negative predictive values

|  | Definition |
| --- | --- |
| Sensitivity | Number of patients with ischemic scar on CMR and CA+ / Number of patients with CA+ : (CMR+CA+) / CA+ |
| Specificity | Number of patients without ischemic scar on CMR nor CA+ / Number of patients without CA+ : (CMR-CA-) / CA- |
| Positive Predictive Value | Number of patients with an ischemic scar on CMR and a CA+ / Number of patients with an ischemic scar on CMR : (CMR+CA+) / CMR+ |
| Negative Predictive Value | Number of patients without ischemic scar on CMR nor CA+ / Number of patients without ischemic scar on CMR : (CMR-CA-) / CMR- |

## Supplemental Table 3. Financial Comparison of Cardiac MRI and Coronary Angiography Procedures

| **Costs (€)** | **Base case value** | **Low estimate** | **High estimate** | **Source** |
| --- | --- | --- | --- | --- |
| **Cost of cardiac magnetic resonance imaging** | | | | |
| Cost of the act | €69 | €69 | €69 | Health insurance |
| Cost of technical charge | €124 | €120 | €139 | Health insurance |
| **Cost of coronary angiography** | | | | |
| Cost of the act | €259 | €259 | €259 | Health insurance |
| **Hospital stay** |  | no stay | severity level 4 |  |
|  | €3,995 | €0 | €77,609 | National hospital information agency |

## Supplemental Table 4. Additional CMR Findings Influencing Patient Management

| **Finding for the 380 cohort patients** | **Number of Patients** | **Percentage of Total Patients (%)** |
| --- | --- | --- |
| **Potential* MINOCA diagnoses** | 78 | 21 |
| **Hypertrophic dilated cardiomyopathy** | 38 | 10 |
| **Myocarditis** | 10 | 3 |
| **Left ventricular non-compaction** | 5 | 1 |
| **Other rare cardiomyopathies** | 5 | 1 |
| **Prevention of unnecessary revascularization** (lack of myocardial viability) | 5 | 1 |
| **Detection of intra-left ventricular thrombus** (missed on echocardiography) | 12 | 3 |

### *There were 78 patients classified as CMR+CA- (CMR+ and not meeting the criteria for "significant CAD" as defined by the study), but these included 35 patients (45%) who were ultimately found to have obstructive coronary lesions (≥50%) in any epicardial coronary segment.Supplemental Table 5 : Interobserver Agreement Between Local Interpretation and Adjudication Committees

|  | **Interpretation by Adjudication Committee** | **Concordant Cases** | **Discordant Cases** | **Kappa (95% CI)** |
| --- | --- | --- | --- | --- |
| **CMR** | Negative | 249 | 30 | 0.64 (0.55–0.73) |
|  | Positive | 76 | 24 |  |
| **CA** | Negative | 328 | 13 | 0.70 (0.59–0.80) |
|  | Positive | 40 | 15 |  |

This table shows the interobserver agreement for cardiac magnetic resonance imaging (CMR) and coronary angiography (CA) between the initial interpretations at the participating centers and those by the independent adjudication committee. Among the patients, 379 had both local and centralized CMR interpretations, and 396 had both local and centralized CA interpretations. Concordant cases indicate agreement between the local and adjudication committee interpretations, while discordant cases indicate disagreement. The Kappa statistic (95% confidence interval) provides a measure of agreement, with values of 0.64 for CMR and 0.70 for CA, indicating substantial agreement.

## Supplemental Table 6 : Coronary artery disease details and revascularization of CA+ patients

| **Variable** | **Total (N=49)** | **CMR+/CA+ (N=28)** | **CMR-/CA+ (N=21)** |
| --- | --- | --- | --- |
| >70% stenosis of the left main coronary artery | 4 ( 8.2%) | 2 ( 7.1%) | 2 ( 9.5%) |
| >70% stenosis of the proximal segment of the left anterior descending coronary artery | 31 ( 63.3%) | 15 ( 53.6%) | 16 ( 76.2%) |
| >70% stenosis of the two epicardial vessels | 22 ( 44.9%) | 15 ( 53.6%) | 7 ( 33.3%) |
| Revascularization | 42 ( 85.7%) | 24 ( 85.7%) | 18 ( 85.7%) |
| Angioplasty | 32 ( 76.2%) | 18 ( 75.0%) | 14 ( 77.8%) |
| CABG | 9 ( 21.4%) | 6 ( 25.0%) | 3 ( 16.7%) |
| Angioplasty+CABG | 1 ( 2.4%) | 0 ( 0.0%) | 1 ( 5.6%) |

## Supplemental Table 7 : Sensitivity of CMR in Predicting CA+ under Various Conditions

| **Scenario** | **Sensitivity (%)** | **Confidence Interval** |
| --- | --- | --- |
| CA Reading Committee opinion for CA+ definition | 59 | 48 – 70 |
| Local interpretations | 62 | 47 - 77 |
| LVEF Subgroup ≤20% | 53 | 28 - 79 |
| LVEF Subgroup 21-35% | 63 | 45 - 81 |
| LVEF Subgroup 36-45% | 43 | 6 - 79 |
| Including LGE, First Pass Perfusion, T1 Mapping | 62 | 47 - 77 |

**Legend:**

CA+: Coronary Artery Disease Positive ; CMR: Cardiac Magnetic Resonance

LGE: Late Gadolinium Enhancement ; LVEF: Left Ventricular Ejection Fraction

Sensitivities are given as percentages with their respective 95% Confidence Intervals.

The table presents the sensitivity of CMR in detecting CAD in different scenarios including various criteria and additional CMR parameters.

### Supplemental Table 8 : Cost-Effectiveness Analysis of CMR-first strategy

3a. Comparing CMR-first and CA-only strategies

|  | CMR- first strategy | CA alone | ICER [IC;95%] |
| --- | --- | --- | --- |
| Diagnostic accuracy | 94.47% | 100,00% | €7,089 **[€-2,445; €21,949]** |
| Average cost/patient | €3,841 | €4,233 |  |

Cost difference : €-392 [95% CI €-122 ; €-1,030]

3b. Comparing CMR-first and "CA and CMR" strategies

|  | CMR- first strategy | CA + CMR | ICER [IC;95%] |
| --- | --- | --- | --- |
| Diagnostic accuracy | 94.47% | 100,00% | €10,597 **[€1,309; €26,754]** |
| Average cost/patient | €3,841 | €4,427 |  |

Cost difference : €-586 [**95% CI** €-71 ; €-1,224]

CA: Coronary Angiography; CMR: Cardiac Magnetic Resonance; ICER: Incremental Cost-Effectiveness Ratio; CI: Confidence Interval.

The reference diagnostic strategy with systematic CA was on average €586 more expensive per patient. ICER emphasizes that each diagnosis of significant coronary artery disease missed with a CMR-first strategy must be balanced against a cost savings of €10,587 compared to routine use of CA alone.

## Supplemental Table 9: The CAMAREC co-investigators

In addition to the authors of the article, the co-investigators of CAMAREC included, in the order of their inscription :

| **Name and Title** | **Location** |
| --- | --- |
| Olivier Nallet, M.D | Montfermeil - Cardiologie |
| Phalla Ou, M.D, PhD | Hôpital Universitaire de Bichat - Radiologie |
| Clément Venner, M.D | Vandoeuvre-lès-Nancy - Institut Lorrain du cœur et des vaisseaux Louis Mathieu - service de cardiologie |
| Atul Pathak, M.D, PhD | Clinique Pasteur - Cardiologie |
| Maxime Benichou, M.D | Hôpital Mercy - Cardiologie |
| Raphaël Aubert, M.D | Hôpital Mercy - Cardiologie |
| Mathieu Becker, M.D | Hôpital Mercy - Cardiologie |
| Marwan Yassine, M.D | Hôpital Mercy - Cardiologie |
| Julien Bayard, M.D | Hôpital Mercy - Cardiologie |
| Julien Bertrand, M.D | Hôpital Mercy - Cardiologie |
| Laura Filippetti, M.D | Vandoeuvre-lès-Nancy - Institut Lorrain du cœur et des vaisseaux Louis Mathieu - service de cardiologie |
| Christine Suty-Selton, M.D | Vandoeuvre-lès-Nancy - Institut Lorrain du cœur et des vaisseaux Louis Mathieu - service de cardiologie |
| Loïc Belle, M.D | Hôpital d'Annecy - Cardiologie |
| Noura Zannad, M.D | Hôpital Mercy - Cardiologie |
| Matthieu Bouygues, M.D | Hôpital d'Annecy - Cardiologie |
| Anis Saib, M.D | Montfermeil - Cardiologie |
| Diana Grigore, M.D | IMM - Cardiologie |
| Selin Atesler, M.D | Hôpital Mercy - Cardiologie |
| Meryl Darlington, MPH | AP-HP, Plateforme de Recherche Clinique en Économie de la Santé (URC-Eco), Hôtel Dieu, Paris, France |
| Jérôme Corré, M.D. | Hôpital Universitaire de La Réunion |
| Reza Rossanaly Vasram, M.D. | Hôpital Universitaire de La Réunion |
| Philippe Boiron, M.D. | Hôpital Universitaire de La Réunion |
| Romain Perrin, M.D. | Hôpital Universitaire de La Réunion |
| Floriane Auclair, M.D. | Hôpital Universitaire de La Réunion |
| Clara Lallemand, M.D. | Hôpital Universitaire de La Réunion |
| Marion Hourqueig, M.D. | Hôpital Universitaire de La Réunion |
| Bertrand Champeau Savon, M.D. | Hôpital Universitaire de La Réunion |

## Supplemental Table 10: Comparative Characteristics of CMR-CA+ and CMR+CA+ Patients

| **Characteristic** | **Total (N=49)** | **CMR-CA+ (N=21)** | **CMR+CA+ (N=28)** | **P-Value** |
| --- | --- | --- | --- | --- |
| **Demographics** | |  |  |  |
| Age, Median [IQR], years | 66.0 [58.0–74.0] | 70.0 [65.0–82.0] | 64.0 [55.0–72.0] | **0.02** |
| Male sex, n (%) | 37 (75.5%) | 14 (66.7%) | 23 (82.1%) | 0.32 |
| **Clinical Characteristics** | |  |  |  |
| Hypertension, n (%) | 22 (44.9%) | 12 (57.1%) | 10 (35.7%) | 0.16 |
| Dyslipidemia, n (%) | 16 (32.7%) | 10 (47.6%) | 6 (21.4%) | 0.07 |
| History of smoking, n (%) | 31 (64.6%) | 13 (61.9%) | 18 (66.7%) | 0.77 |
| Diabetes, n (%) | 17 (34.7%) | 5 (23.8%) | 12 (42.9%) | 0.23 |
| Family history of cardiovascular disease, n (%) | 7 (14.3%) | 3 (14.3%) | 4 (14.3%) | 1.00 |
| Peripheral artery disease, n (%): | 7 (14.3%) | 3 (14.3%) | 4 (14.3%) | 1.00 |
| BMI, Median [IQR] | 26.3 [22.8–29.7] | 24.8 [21.5–29.7] | 26.8 [23.6–29.7] | 0.36 |
| Atrial fibrillation, n (%): | 4 (8.2%) | 2 (9.5%) | 2 (7.1%) | 1.00 |
| Presence of cardiotoxicity (HIV or alcoholism), n (%): | 13 (27.1%) | 5 (23.8%) | 8 (29.6%) | 0.75 |
| **Laboratory Findings** | |  |  |  |
| Troponin I, Median [IQR], µg/L | 0.3 [0.1–0.5] | 0.1 [0.0–0.3] | 0.4 [0.2–0.7] | **0.02** |
| Troponin T, n (%) | 7 (15.6%) | 2 (11.8%) | 5 (17.9%) | 0.69 |
| **Imaging Features** | |  |  |  |
| Centromycocardial LGE, n (%) | 8 (16.3%) | 6 (28.6%) | 2 (7.1%) | 0.06 |
| Subepicardial LGE, n (%) | 6 (12.2%) | 4 (19.0%) | 2 (7.1%) | 0.38 |
| Presence of one or more T1 mapping abnormalities, n (%) | 33 (82.5%) | 14 (73.7%) | 19 (90.5%) | 0.23 |
| LVEDD (median [IQR]), mm | 60.0 [54.0–64.0] | 60.0 [55.0–65.0] | 59.0 [54.0–63.0] | 0.76 |
| LVESD (median [IQR]), mm | 52.0 [45.0–56.0] | 47.0 [43.0–55.0] | 53.0 [46.0–58.0] | 0.32 |
| LVEDV (median [IQR]), mL | 171.5 [117.0–210.0] | 150.0 [114.0–222.0] | 180.0 [125.0–205.5] | 0.36 |
| **Right Ventricular Function** | | |  |  |
| Dilatation of RV, n (%) | 9 (18.4%) | 3 (14.3%) | 6 (21.4%) | 0.90 |
| Dysfunction of RV, n (%) | 15 (30.6%) | 7 (33.3%) | 8 (28.6%) | 0.59 |
| **ECG Findings** | |  |  |  |
| Sinus rhythm, n (%) | 40 (81.6%) | 17 (81.0%) | 23 (82.1%) | 1.00 |
| Complete right bundle branch block, n (%) | 3 (6.4%) | 3 (15.0%) | 0 (0.0%) | 0.07 |
| Presence of significant Q wave, n (%) | 10 (21.3%) | 4 (21.1%) | 6 (21.4%) | 1.00 |
| Negative T wave/ST anomaly, n (%) | 23 (48.9%) | 10 (52.6%) | 13 (46.4%) | 0.77 |
| **Location of the Lesions** | |  |  | 0.12 |
| LAD (2nd segment) + RCA, n (%) | 17 (34.7%) | 5 (23.8%) | 12 (42.9%) |  |
| Proximal LAD (1st segment), n (%) | 13 (26.5%) | 9 (42.9%) | 4 (14.3%) |  |
| **Number of Vessels Involved** | | |  | 0.03 |
| 1 Vessel, n (%) | 10 (20.4%) | 6 (28.6%) | 4 (14.3%) |  |
| 2 Vessels, n (%) | 19 (38.8%) | 11 (52.4%) | 8 (28.6%) |  |
| 3 Vessels, n (%) | 20 (40.8%) | 4 (19.0%) | 16 (57.1%) |  |

This table compares demographic, clinical, laboratory, imaging, and ECG characteristics between patients classified as CMR-CA+ (false negatives) and CMR+CA+ (true positives). Continuous variables are expressed as median [interquartile range], and categorical variables are presented as absolute numbers (percentages). P-values were calculated to compare the two groups using appropriate statistical tests. Abbreviations: BMI (Body Mass Index), LGE (Late Gadolinium Enhancement), LVEDD (Left Ventricular End-Diastolic Diameter), LVESD (Left Ventricular End-Systolic Diameter), LVEDV (Left Ventricular End-Diastolic Volume), RV (Right Ventricle), LAD (Left Anterior Descending Artery), RCA (Right Coronary Artery). Significant P-values (<0.05) are indicated in bold.

## Supplemental Figure 1. Examples of 4 patients with significant CAD on CA without subendocardial late gadolinium enhancement on CMR


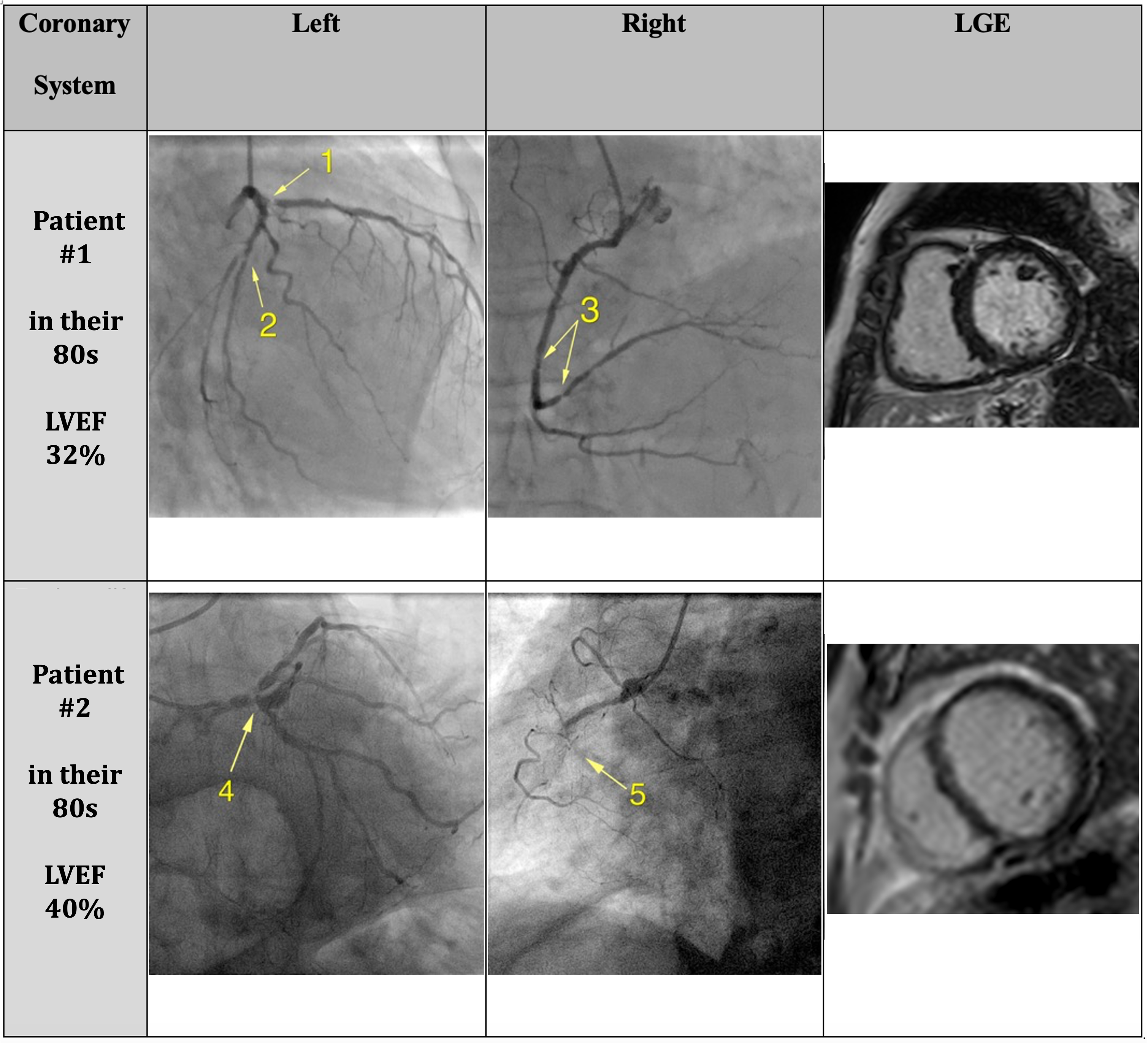


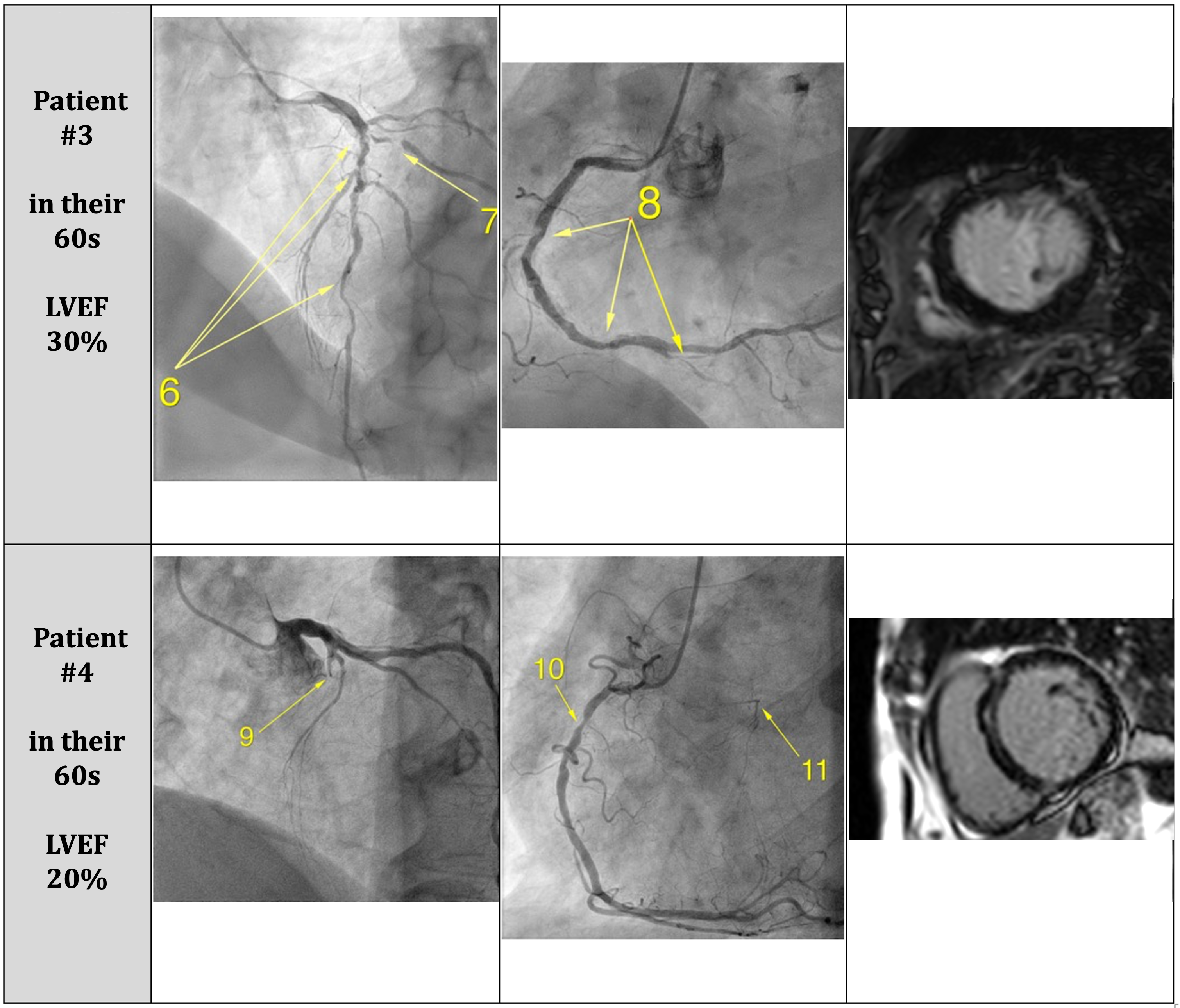


**Figure legend**: **1:** significant lesion of ostial Left Anterior Descending artery (LAD), **2:** significant lesion of mid Left Circumflex artery (LCx), **3:** severe stenosis of both mid and distal Right Coronary Artery (RCA), **4:** critical lesion of left main, **5:** chronic total occlusion of proximal RCA, **6:** Long diffuse and severely calcified lesions of proximal and mid LAD, **7:** significant lesion of first diagonal branch, **8:** multiples lesions of mid and distal RCA, **9:** chronic total occlusion of proximal LAD, **10:** significant stenosis of proximal RCA, **11:** collaterals toward proximal LAD

### Supplemental Figure 2. Cost-Effectiveness Analysis of CMR-First Strategy

2a. CMR-first strategy cheaper but less effective than CA-only strategy
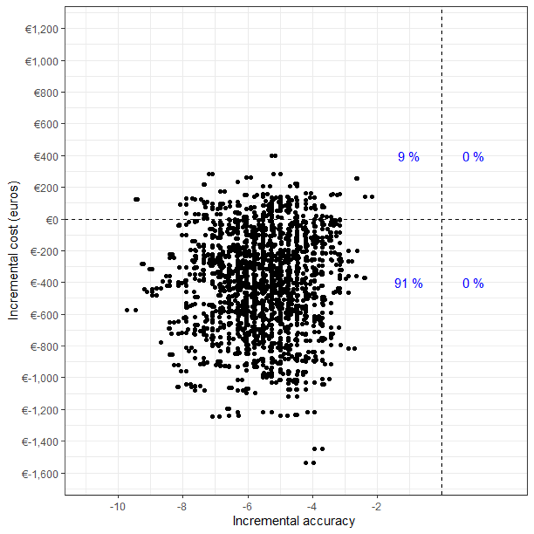


2b. CMR-first strategy cheaper but less effective than “CA and CMR” strategy
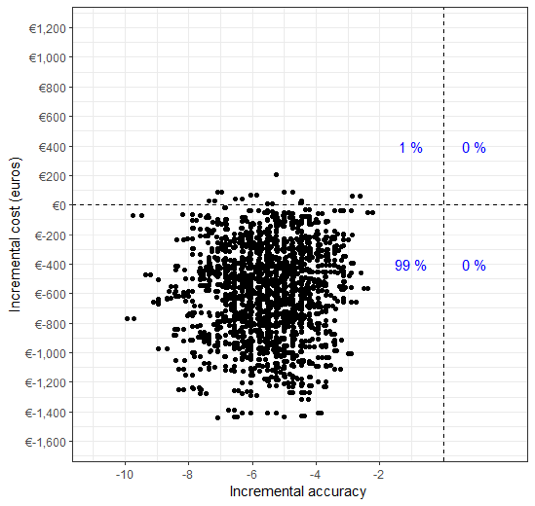


**Figure legend**: Comparative cost-effectiveness scatter plots from 1,000 bootstrap replicates of CAMAREC data.

3a: Demonstrates a CMR-first strategy versus CA alone, with the former being on average €392 less costly per patient, offering a 91% chance of being more cost-effective despite lower diagnostic accuracy.

3b: Compares a CMR-first strategy to the combined 'CA and CMR' approach, with the CMR-first being on average €586 less costly per patient, and a 99% chance of being more cost-effective, though with reduced diagnostic accuracy.

*CA: coronary angiography; CMR: cardiac magnetic resonance. Costs in Euros (€) and diagnostic accuracy as a percentage (%).
